# Systemic antibiotics for preventing ventilator-associated pneumonia in comatose patients: a systematic review and meta-analysis

**DOI:** 10.1186/s13613-017-0291-4

**Published:** 2017-06-15

**Authors:** Cássia Righy, Pedro Emmanuel Americano do Brasil, Jordi Vallés, Fernando A. Bozza, Ignacio Martin-Loeches

**Affiliations:** 1 National Institute of Infectious Disease Evandro Chagas, Oswaldo Cruz Foundation (FIOCRUZ), Rio de Janeiro, Brazil; 2 ICU, Paulo Niemeyer Brain Institute, Rio de Janeiro, Brazil; 3 CIBER Enfermedades Respiratorias (CIBERES), Barcelona, Spain; 4 Critical Care Center, CIBER Enfermedades Respiratorias, Hospital Sabadell, Sabadell, Spain; 5 IDOR, D’Or Institute for Research and Education, Rio de Janeiro, Brazil; 6 Department of Clinical Medicine, Trinity Centre for Health Sciences, Multidisciplinary Intensive Care Research Organization (MICRO), Wellcome Trust, HRB Clinical Research, St James’s University Hospital Dublin, Dublin, Ireland; 7 Irish Centre for Vascular Biology (ICVB), Dublin, Ireland

**Keywords:** Systematic review, Meta-analysis, Ventilator-associated pneumonia, Coma

## Abstract

**Background:**

Early-onset ventilator-associated pneumonia (EO-VAP) is the leading cause of morbidity and mortality in comatose patients. However, VAP prevention bundles focus mainly on late-onset VAP and may be less effective in preventing EO-VAP in comatose patients. Systemic antibiotic administration at the time of intubation may have a role in preventing EO-VAP. Therefore, we evaluated the effectiveness of systemic antibiotic administration in VAP prevention in comatose patients through a systematic review and meta-analysis.

**Methods:**

We searched for studies published through December 2015 that evaluated systemic antibiotic prophylaxis in comatose patients. Two authors independently selected and evaluated full-length reports of randomized clinical trials or prospective cohorts in patients aged >16 years that evaluated the impact of systemic antibiotics at the time of intubation on EO-VAP compared to placebo or no prophylaxis. The outcome variables were the incidence of EO-VAP, the duration of mechanical ventilation, ICU length of stay, and ICU mortality.

**Results:**

We identified 10,988 citations, yielding 26 articles for further analysis; three studies with 267 patients were finally analyzed. Most patients (*n* = 135) were comatose due to head trauma. Systemic antibiotic administration was associated with decreased incidence of EO-VAP (RR 0.32; 95% CI 0.19–0.54) and shorter ICU LOS (standardized mean difference −0.32; 95% CI −0.56 to −0.08), but had no effect on mortality (RR 1.03; 95% CI 0.7–1.53) or duration of mechanical ventilation (standardized mean difference −0.16; 95% CI −0.41 to 0.08).

**Conclusions:**

Antibiotic prophylaxis in comatose patients reduced the incidence of EO-VAP and decreased the ICU stay slightly. Future trials are needed to confirm these results.

## Background

Ventilator-associated pneumonia (VAP) is a frequent cause of morbidity and mortality in comatose patients. In this population, pneumonia usually occurs within the first four days of mechanical ventilation and is termed early-onset pneumonia (EO-VAP) [1]. The incidence of EO-VAP ranges from 21 to 60% [2, 3] in critically ill patients with traumatic brain injury (TBI) and is about 48% in those with subarachnoid hemorrhage. In a mixed population of patients in coma due to various causes, EO-VAP accounted for 70% of all cases of pneumonia [4]. In neurosurgical patients, the incidence of VAP peaks in the first three days after admission [5]. Pneumonia is associated with higher mortality in acute neurological patients [6]; a recent meta-analysis by the Cochrane Group found that pneumonia in stroke patients is associated with mortality (OR 3.62) [7]. Jovanovic et al. [8] found that VAP was associated with higher and earlier mortality in comatose patients with TBI.

The predominance of EO-VAP in comatose patients is in striking contrast to general critical care patients, in whom accounts for 62–73% of all cases of VAP are late onset [9, 10]. Many risk factors are related to the increased incidence of EO-VAP in brain-injured patients. Massive or microbronchoaspiration, leakage of colonized subglottic secretions around the cuff of the endotracheal tube, and brain injury-induced immunosuppression may all play significant roles [11]. Moreover, it is not always feasible to implement VAP prevention bundles in brain-injured patients [12], and preventive measures that are effective for late-onset VAP might not be effective for EO-VAP [13]. Thus, alternative prophylactic measures should be explored in comatose patients.

Among other measures, antibiotic administration at the time of intubation seems a reasonable alternative. Systemic antibiotics have been reported to protect against EO-VAP [14]. However, the role of antibiotic prophylaxis in comatose patients remains unclear. Therefore, we performed a systematic review and meta-analysis of prospective studies to answer the following question: Is the administration of systemic antibiotics at the time of intubation superior to placebo or no prophylaxis in preventing VAP and decreasing all-cause mortality reduction in comatose patients?

## Methods

### Data sources and study selection

Following the methodological recommendations of the Cochrane Collaboration and the PRISMA statement [15], two authors (CR and IML) independently searched PubMed and the Cochrane Library (2015) for the terms aspiration pneumonia, pneumonia, ventilator-associated pneumonia (VAP), coma, altered level of consciousness, and depressed level of consciousness, cross-referenced to the terms antibiotic prophylaxis, and preemptive antibiotic therapy. The search strategy performed was the following:

First Search: #1

Search ((((((((((aspiration pneumonia[MeSH Terms]) OR “pneumonia”[MeSH Terms]) OR “pneumonia, ventilator associated”[MeSHTerms]) OR respiratory infections[MeSH Terms]) AND coma[MeSH Terms])OR altered level of consciousness[MeSH Terms]) OR depressed level of consciousness[MeSH Terms]) OR consciousness disorder[MeSH Terms]) OR consciousness, loss of[MeSH Terms]) AND antibiotic prophylaxis[MeSHTerms]) OR antibiotic premedication[MeSH Terms].

Second Search: #2

Search (((((((((“aspiration pneumonia”[Title/Abstract]) OR”pneumonia”[Title/Abstract]) OR “ventilator associated pneumonia”[Title/Abstract]) OR “respiratory infection”[Title/Abstract])AND “coma”[Title/Abstract]) OR “depressed level of consciousness”[Title/Abstract]) AND “antibiotic prophylaxis”[Title/Abstract]) OR “antibiotic premedication”[Title/Abstract]) OR “preemptive antibiotic treatment”[Title/Abstract]) OR “preemptive antibiotic therapy”[Title/Abstract].

Third Search:

#1 OR #2

Then, we manually searched personal files for full-length articles published in peer-reviewed journals by May 12 (2017). We selected the inclusion criteria for articles using the PICO approach. The inclusion criteria were: (1) clinical trials or prospective cohorts; (2) population analyzed—adult (>18 years) comatose patients; (3) intervention—systemic antibiotic prophylaxis at the time, or just before orotracheal intubation; (4) control group—patients who did not receive antibiotics for intubation; and (5) outcome—studies that evaluated VAP incidence, ICU and hospital mortality, as well as length of hospital stay and length of mechanical ventilation. We excluded studies that did not report enough data to estimate the odds ratio (OR) or relative risk (RR) and their variance.

Two authors (CR and IML) screened citations and articles identified by the initial search, selecting potentially relevant titles, reviewing their abstracts, and determining whether the articles met the inclusion criteria. We also searched the reference lists in the selected articles to look for any study that was not identified in the original search. The protocol was published in the International Prospective Register of Systematic Reviews (PROSPERO identifier: CRD42016033698).

### Data extraction and study quality assessment

Two authors (CR and IML) independently abstracted data from the selected articles, recording the following information, when available:

- Study characteristics (study location, period of enrollment, criteria for patient enrollment, number of patients enrolled, duration of follow-up);
- Study design;
- Patients’ characteristics (age, sex, mechanical ventilation, disease severity, cause of coma, and Glasgow Outcome Scale);
- VAP definition (early and late onset);
- Antibiotic therapy and controls;
- Outcomes (incidence of VAP (early and late); ICU and hospital mortality; duration of mechanical ventilation; ICU and hospital length of stay).

Any discrepancies were resolved by discussion among authors (CRS, IML, FAB). If data were not reported, we planned to contact first or senior authors by email.

To assess the methodological quality of the studies included, we used the Cochrane Risk of Bias Tool (for RCT) [16] and the Newcastle-Ottawa Score, for observational studies.

### Outcomes

The main outcomes of interest were incidence of VAP (early and late) and ICU and hospital mortality. The secondary outcomes were ICU and hospital length of stay as well as duration of mechanical ventilation.

### Patient involvement

This review and meta-analysis did not involve patients directly.

### Statistical analysis

We compared patients’ characteristics and outcomes between the group of patients who received antibiotic prophylaxis and those who did not (control group). Primary outcome variables were the incidence of EO-VAP and ICU mortality; secondary outcome variables were ICU length of stay and duration of mechanical ventilation.

Primary outcome variables are reported as relative risks (RR) with their corresponding 95% confidence intervals (CI), analyzed with the Mantel–Haenszel fixed-effects method. Secondary outcome variables are reported as standardized mean differences (SMD) with their respective 95% CI. To assess the impact of heterogeneity across studies on the meta-analysis, we used the *I* ^2^ statistic, which reflects the amount of heterogeneity between studies over and above the sampling variation and is robust to the number of studies and choice of effect measure. We used the R statistical package for all analyses.

## Results

**Fig. 1:**
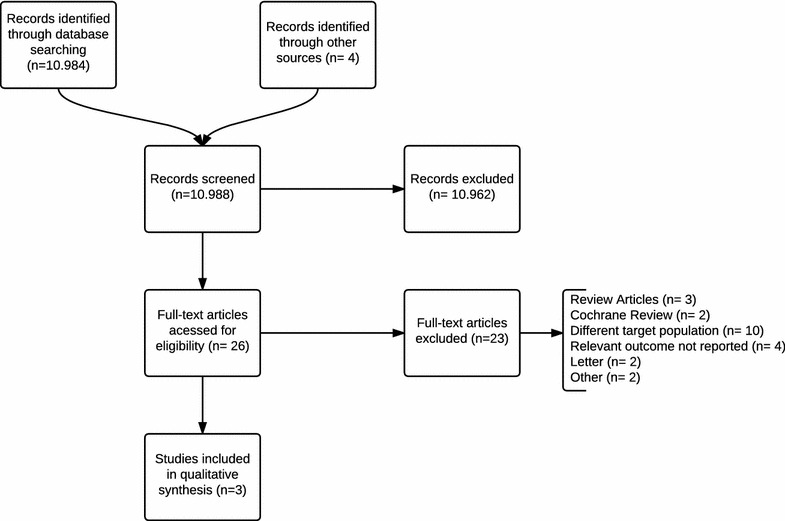
Selection of studies on antibiotic use for VAP prevention in comatose patients

The literature search produced 11,340 citation titles, yielding 26 articles for detailed analysis; three studies including a total of 267 patients met the inclusion criteria and were included in the systematic review (Fig. 1).

### Definitions

All studies defined EO-VAP as pneumonia developed within the first four days of mechanical ventilation. Clinical criteria for VAP definition varied among the studies; however, all studies required a microbiological confirmation of pneumonia—either by bronchoalveolar lavage (BAL) or by protected brush sampling or by tracheal aspirate. The definition of coma also varied—Vallés et al. [17] and Acquarolo et al. [18] defined as a Glasgow Coma Scale (GCS) ≤ 8 and Sirvent et al. [4] defined it as a GCS ≤ 12. The definitions are summarized in Table 1.

**Table 1:** Studies’ characteristics

	Sirvent JM et al.		Acquarolo A et al.		Vallés J et al.	
Year published	1997		2007		2013	
Country	Spain		Italy		Spain	
Study design	RCT		RCT		Prospective study with historical control or non-randomized control	
Inclusion criteria	Head injury or coma due to stroke or surgery for space occupying lesions with Glasgow ≤ 12		Adults, comatose patients (GCS ≤ 8) in mechanical ventilation		Adults, comatose patients (GCS ≤ 8) in mechanical ventilation	
Tested antibiotic	Cefuroxime		Ampicillin–sulbactam		Ceftriaxone or ertapenem or levofloxacin	
Antibiotics used in control group?^b^	Yes		Yes		No	
	Intervention group	Control group	Intervention group	Control group	Intervention group	Control group
Number of subjects	50	50	19	19	71	58
Age (mean ± SD), years	42 ± 20	37 ± 21	54.8 (18.0)^a^	54.6 (17.7)^a^	56 ± 19	59 ± 16
Male gender, *n* (%)	34 (68%)	40 (80%)	13 (68.4%)	12 (63.2%)	48 (67.6%)	43 (74.1%)
Glasgow Coma Scale (mean ± SD)	7.5 ± 2.4	8.0 ± 1.8	5 (3–7)^a^	5 (4–7)^a^	5 ± 2	5 ± 2
APACHE II (mean ± SD)	14 ± 5	13 ± 5	20 (17–24)^a^	22 (18–23)^a^	17 ± 7	18 ± 7
Early VAP, *n* (%)	8 (16%)	18 (36%)	4 (21%)	11 (57.8%)	2 (2.8%)	13 (22.4%)
Late VAP, *n* (%)	4 (8%)	7 (14%)	10 (episodes)	9 (episodes)	6.5 (incidence/1000 days MV)	5.3 (incidence/1000 days MV)
Total VAP, *n* (%)	12 (24%)	25 (50%)	14 (episodes)	20 (episodes)	10.8 (incidence/1000 days MV)	28.4 (incidence/1000 days MV)
Duration of mechanical ventilation (mean ± SD), days	4.6 ± 1.5	4.4 ± 2.1	9.9 (6.9)^a^	10.6 (9.4)^a^	6.4 ± 6.5	9.7 ± 9.6
ICU LOS (mean ± SD), days	13 ± 8	16 ± 11	12.8 (8.7)^a^	12.6 (9.7)^a^	9.7 ± 9.8	14.9 ± 13.9
Hospital LOS (mean ± SD), days	27 ± 16	28 ± 13	Not informed	Not informed	17.5 ± 17.7	23.5 ± 24.3
ICU mortality, *n* (%)	10 (20%)	7 (14%)	7 (36.8%)	8 (42.1%)	21 (29.6%)	18 (31%)

*RCT* randomized controlled trial, *VAP* ventilator-associated pneumonia, *LOS* length of stay, *APACHE II* Acute Physiology and Chronic Health Evaluation II, *MV* mechanical ventilation

^a^Median (interquartile range)

^b^Antibiotics used mainly as surgical prophylaxis

### Characteristics of the studies included

The meta-analysis included two randomized clinical trials and one prospective observational cohort with a non-randomized historical control group. Table 2 provides detailed information about the three studies. Study populations ranged from 38 to 129 patients. The most common cause of coma was head trauma (*n* = 135), followed by stroke (*n* = 49) and cardiac arrest (*n* = 37). All studies evaluated EO-VAP, defined as VAP acquired within four days after intubation for mechanical ventilation. All studies reported short-term outcomes (ICU mortality, duration of mechanical ventilation, and ICU LOS). Two studies also evaluated hospital mortality. Table 3 provides details about the Jadad Scale evaluation of the methodological quality of the studies. There was no heterogeneity among the studies in the main outcomes.

**Table 2:** Summary of the quality evaluation by Jadad Scale of clinical trials of antibiotic prophylaxis in comatose patients

	Randomization	Blinding	Description of withdrawals and dropout	Score
Sirvent JM	2	0	1	3
Acquarolo A	2	2	1	5
Vallés J	0	0	1	1

**Table 3:** VAP microbiology

	Early VAP	Late VAP
Sirvent et al.	*S. aureus*—14	Enterobacter—1
	*H. influenzae*—11	Serratia—2
	*Strep pneumoniae*—1	Proteus—1
		*P. aeruginosa*—4
		Acinetobacter sp—3
Vallés et al.	*S. aureus*—3	E cloacae—3
	Anaerobes—1	*S. aureus*—1
	Mixed flora—1	*P. aeruginosa*—3
		*Streptococcus* sp—1
Total	*S. aureus*—17	*P. aeruginosa*—7
	*H. influenza*—11	E cloacae/Acinetobacter sp—3 each
	*S. pneumoniae*/Anaerobes/mixed flora—1 each	Serratia—2
		Enterobacter/Proteus/*S. aureus*/*Streptococcus* sp—1 each

### Main outcomes

**Fig. 2:**
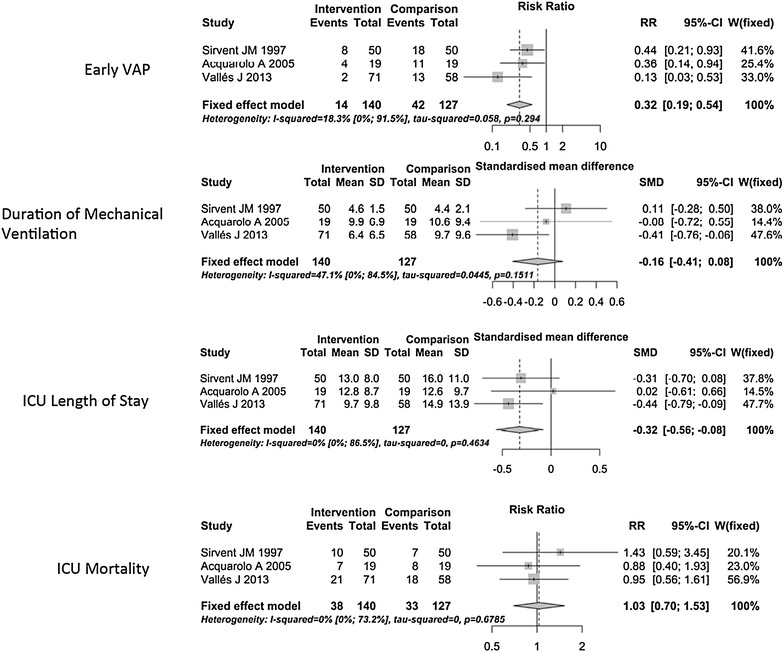
Impact of antibiotic prophylaxis on early VAP, ICU mortality, duration of mechanical ventilation, and ICU length of stay

Figure 2 shows the association between systemic antibiotic administration and the outcomes of interest. The RR of EO-VAP was 0.32 (95% CI 0.19–0.54, *p* < 0.01), favoring the intervention group, which suggests a protective effect, and the RR of ICU mortality was 1.03 (95% CI 0.7–1.53, *p* = 0.88), showing no protective effect.

Antibiotic administration did not affect the duration of mechanical ventilation (SMD: −0.16; 95% CI −0.41–0.08, *p* = 0.18), but decreased ICU LOS slightly (SMD: −0.32; 95% CI −0.56 to −0.08, *p* < 0.01), indicating that antibiotic prophylaxis reduced the ICU stay by 9 h on average.

## Discussion

Antibiotic prophylaxis seems effective in preventing EO-VAP in a mixed cohort of comatose patients and may also decrease the ICU LOS slightly; however, it had no effect on the length of mechanical ventilation or ICU mortality.

One explanation for the decrease in the incidence of EO-VAP in comatose patients receiving antibiotic prophylaxis is the reduction of bacterial inoculum in the lungs. After brain injury, many concurrent phenomena act to increase the bacterial burden within the alveolar space: micro- or macroaspiration, brain-induced immunosuppression, or even increased capillary leakage from sympathetic overstimulation [19]. Antibiotic administration may prevent the propagation of bacteria into the lung, thereby preventing EO-VAP, whereas many traditional prophylactic measures included in the VAP bundle may be less effective in brain-injured patients.

EO-VAP is frequent in critically ill acute neurological patients. Although we found no impact of antibiotic prophylaxis on ICU mortality, preventing EO-VAP may reduce overall antibiotic use and costs, improve functional prognosis, and indirectly decrease long-term mortality. Finlayson et al. [20] showed that, in patients with ischemic stroke, pneumonia is associated with higher 30-day and 1-year mortality, as well as with a poorer functional outcome. While mortality from infection is estimated to account for up to 30% of stroke deaths and infection is an independent predictor of neurological deterioration, patients that have a decrease in EO-VAP did not show significantly different mortality [21]; this kind of cases are specially complex and might present some complications especially common in this subset of patients. In addition, while the mortality represents a very robust outcome, we consider that mortality could be analyzed cautiously just because of the impact of this intervention. Moreover, infection is the primary cause of readmission after stroke [22]. Hospital-acquired pneumonia is independently associated with poor functional outcome up to 5 years after TBI [23].

Our meta-analysis also found that antibiotic prophylaxis decreased ICU LOS slightly, possibly due to the decrease in EO-VAP. Just as treating tracheobronchitis can lead to a reduction in VAP and consequent reduction in ICU LOS [24, 25], prophylaxis against EO-VAP may have an impact in reducing ICU LOS and may also affect functional outcomes. However, none of the studies included in the meta-analysis was designed to evaluate functional outcome, so we could not assess the effect of antibiotic prophylaxis on long-term prognosis.

Whether antibiotic prophylaxis induces bacterial resistance is a well-founded concern. An association between broad-spectrum systemic antibiotics and the development of antibiotic resistance was pointed out a decade ago [26]. Depuydt et al. [27] showed VAP involving multidrug-resistant pathogens was associated with higher mortality and that the risk of developing VAP involving multiresistant bacteria was associated with previous antibiotic use. These findings have led to the development of antibiotic stewardship programs that minimize antibiotic exposure to diminish resistance to antibiotics and improve outcomes [28].

However, the use of one- or two-dose prophylactic antibiotic regimens may not be as deleterious to the patient and ICU ecology as prolonged, inadequate antibiotic administration. In a landmark randomized controlled trial, Chastre et al. [29] showed that multidrug-resistant pathogens emerged less frequently in recurrent infections developing in patients assigned to an 8-day course of antibiotic than in those developing in patients assigned to a 15-day course (42.1 vs. 62.3%). In neutropenic patients, quinolone prophylaxis was not associated with increased antimicrobial resistance [30], and in cardiac surgery patients, antibiotic resistance was associated only with antibiotic prophylaxis for more than 48 h [31]. Although our meta-analysis was not planned to analyze this issue, none of the studies included reported any increase in multidrug-resistant pathogens. This finding suggests that very short antibiotic regimens may not lead to greater resistance, but this hypothesis must be confirmed in future trials.

Antibiotic prophylaxis is a simple and cheap measure that can be easily reproduced all around the world. Other prophylactic measures have not proven effective. Corticosteroid administration was not effective in preventing nosocomial pneumonia in TBI patients, although the overall incidence of pneumonia in this study was lower than expected [32]. The effectiveness of beta-blockers [33, 34], or statins [35, 36], in preventing pneumonia in stroke patients is controversial and must be tested in future trials.

This meta-analysis has some limitations. First and most importantly, numbers of studies are low, which has consequences for the interpretation of the data and may amplify a hypothetically minor impact on pneumonia prevention. We would, however, highlight our surprise in such an easy intervention and the low number of studies conducted when compared to the literature of more complex interventions [37, 38]. Based on the low number of patients in each subgroup and the lack of individual information, we considered that more exploratory analyses such as: (1) dose of antibiotic; (2) time of initiation; and (3) type of antibiotic would increase the heterogeneity and would not allow robust conclusions. Moreover, most patients were admitted to intensive care for TBI, and this patient mix might limit the generalizability of our results. Finally, the broad-spectrum antibiotic regimens tested varied among the different studies. Despite the common goal of preventing EO-VAP, these differences in antibiotic use, dosage, and timing of administration may preclude the analysis of their impact as a single group. However, the results of this meta-analysis support the hypothesis that antibiotic prophylaxis reduces EO-VAP without increasing antimicrobial resistance.

## Conclusion

This meta-analysis found that antibiotic prophylaxis in comatose patients reduced the incidence of EO-VAP and decreased ICU LOS slightly. However, a larger randomized trial focusing on measuring both the decrease in the incidence of EO-VAP and possible improvements in long-term functional outcomes is needed to confirm these findings. The 2005 ATS guidelines on hospital-acquired pneumonia concluded that although administering antibiotics at the time of intubation may prevent EO-VAP, its routine use could not be recommended until more evidence was available [39]. The current IDSA/ATS guidelines do not make any recommendation regarding antibiotic prophylaxis against EO-VAP [40]. We were surprised that a non-complex intervention has been not widely studied. We think that the results of this meta-analysis strongly support undertaking new clinical trials in the incidence of EO-VAP in other subsets of critically ill patients and possible improvements in long-term functional.
